# Six-year experience of Australia’s first dedicated cancer of unknown primary clinic

**DOI:** 10.1038/s41416-023-02254-6

**Published:** 2023-05-24

**Authors:** Arielle van Mourik, Gina Tonkin-Hill, John O’Farrell, Shohei Waller, Lavinia Tan, Richard W. Tothill, David Bowtell, Stephen Fox, Andrew Fellowes, Clare Fedele, Penelope Schofield, Tharani Sivakumaran, Hui-Li Wong, Linda Mileshkin

**Affiliations:** 1 Department of Medical Oncology, Peter MacCallum Cancer Centre, Melbourne, VIC Australia; 2 The Sir Peter MacCallum Department of Oncology, The University of Melbourne, Parkville, VIC Australia; 3 Department of Clinical Pathology and Centre for Cancer Research, The University of Melbourne, Parkville, VIC Australia; 4 Cancer Research Division, Peter MacCallum Cancer Centre, Melbourne, VIC Australia; 5 Department of Pathology, Peter MacCallum Cancer Centre, Melbourne, VIC Australia; 6 CSL Innovation, Melbourne, VIC Australia; 7 Department of Psychology, and Iverson Health Innovation Research Institute Swinburne University, Melbourne, VIC Australia; 8 Behavioural Sciences Unit, Health Services Research and Implementation Sciences, Peter MacCallum Cancer Centre, Melbourne, VIC Australia

**Keywords:** Cancer of unknown primary, Cancer genomics

## Abstract

**Background:**

Diagnosis and management of cancers of unknown primary (CUP) remain challenging. This study examines the referral patterns, management and outcomes of patients referred to Australia’s first dedicated CUP clinic.

**Methods:**

Retrospective medical record review was conducted for patients seen at the Peter MacCallum Cancer Centre CUP clinic between July 2014 and August 2020. Overall survival (OS) was analysed for patients with a CUP diagnosis where treatment information was available.

**Results:**

Of 361 patients referred, fewer than half had completed diagnostic work-up at the time of referral. A diagnosis of CUP was established in 137 (38%), malignancy other than CUP in 177 (49%) and benign pathology in 36 (10%) patients. Genomic testing was successfully completed in 62% of patients with initial provisional CUP and impacted management in 32% by identifying a tissue of origin or actionable genomic alteration. The use of site-specific, targeted therapy or immunotherapy was independently associated with longer OS compared to empirical chemotherapy.

**Conclusion:**

Our specialised CUP clinic facilitated diagnostic work-up among patients with suspected malignancy and provided access to genomic testing and clinical trials for patients with a CUP diagnosis, all of which are important to improve outcomes in this patient population.

## Background

Patients with carcinoma of unknown primary site (CUP) present with metastatic disease for which an anatomical primary tumour cannot be identified despite standardised work-up, and account for 1–2% of advanced malignancies worldwide [1]. However, autopsy studies have demonstrated small invasive primary cancers in up to 73% of CUP patients, with additional cases potentially undetected considering the thousands of tissue sections required to detect a very small cancer [2]. In Australia, 2380 CUP cases were diagnosed in 2019 with a rate of 7.4/100,000 people [3]. Men and women are affected equally, and the average age at diagnosis is 60–75 years [3].

Presentation of CUP varies widely, from solitary sites of disease to multiple metastases. Frequently affected sites include lymph nodes, bones, liver and lungs [4, 5]. Risk factors may include current or former smoking, whilst associations have also been made with low educational attainment, older age, and female sex [6]. In addition, some studies have identified a potential familial predisposition to CUP with family members being at increased risk of lung, pancreatic and CUP malignancies [7].

International guidelines exist to direct the diagnostic approach to patients with potential CUP, and include a thorough history and examination, basic blood analysis, computed tomography of chest, abdomen and pelvis (CTCAP), tissue biopsy with immunohistochemistry staining and mammogram in females [8–10]. Further investigations depend on the likely primary site, such as alpha fetoprotein and human chorionic gonadotropin in men with midline disease, prostate specific antigen in men with osteoblastic bone metastases, and endoscopy in patients with clinical features or immunohistochemistry suggestive of gastrointestinal origin. The cost of diagnostic work-up in this group of patients is typically higher than in those patients with carcinomas of known primaries [11], as is the emotional burden and impact on quality of life for affected patients and families [12, 13].

CUP malignancies are categorised as either favourable or unfavourable based on histological subtype, the pattern of disease, availability of specific treatment, and estimated prognosis [8]. Favourable CUP typically includes histological subtypes for which effective systemic therapies are available, such as colon, breast, ovary and prostate cancers. With the increasing use of targeted therapies and immune checkpoint inhibitors, improved outcomes are being observed across traditionally poor prognosis subtypes, such as lung and renal cancers [14]. Hence, the European Society of Medical Oncology (ESMO) classification of favourable CUP subtypes has recently been updated to include renal-like CUP [15]. In patients with unfavourable subtypes of CUP, the mainstay of treatment remains empirical platinum-based chemotherapy with a median overall survival (OS) of ~6 months, while patients with favourable subtypes typically have median OS of 12–36 months [16].

Genomic testing is playing an increasingly prominent role in the work-up of CUP with the dual aims of identifying a tissue of origin, and potentially targetable molecular alterations [17]. Although the use of site- or molecular-directed treatment is not yet proven to improve OS, up to a third of patients may have an actionable molecular alteration which can be targeted with a specific medication [18]. Across Australia, genomic testing is not publicly funded for CUP, so patients can only access this through clinical trials or by self-funding at a cost of several thousand dollars.

A dedicated Carcinoma of Unknown Primary Clinic was established at the Peter MacCallum Cancer Centre in 2012, allowing patients to be systematically assessed and facilitating access to genomic testing and clinical trials. This is the first dedicated CUP clinic in Australia. In this retrospective study, we describe our experience with patients seen in the CUP clinic with three key aims. The first was to describe the referral and diagnostic work-up patterns of patients seen in the clinic. Secondly, we sought to determine the proportion of patients in whom a primary malignancy or tissue of origin could be identified. The third aim was to describe the relationship between treatments received, CUP subtype and outcomes of patients with a CUP diagnosis.

## Methods

### Setting

Peter MacCallum Cancer Centre is a comprehensive cancer centre located in metropolitan Melbourne, Australia. The CUP clinic was established in 2012 and now runs once a week, with two medical oncologists and one medical oncology fellow seeing patients. Referrals are triaged by either the consultants or fellow and are accepted from primary care and emergency physicians, community oncologists, medical specialists and surgeons around the country. Patient referrals are accepted both with and without a biopsy, if there is likely malignancy of unknown origin (MUO) based on imaging evidence to suggest metastatic disease without an obvious primary identified during initial work-up [10]. Referrals are also accepted for access to genomic testing via clinical trials including the SUPER (Solving Unknown Primary cancER) study [19]. Radiation oncology and palliative care services are readily accessible to receive referrals if required.

### Study design and population

After gaining ethics approval (HREC QA/69106/PMCC-2020), we conducted a retrospective medical record review of all patients booked to the CUP clinic at Peter MacCallum Cancer Centre from 1 July 2014 to 3 August 2020. Patients with a known primary tumour booked to see a particular physician for a reason other than CUP were excluded. Demographic, disease, treatment and outcome data were collected for the remaining patients. Waiver of consent was approved by the institutional ethics committee due to the low-risk nature of this study.

### Data collection

Data were extracted from each patient’s medical record. Demographic data included age, sex, ECOG performance status, Charlson Comorbidity Index (excluding current malignancy and adjusted for age) [20], smoking history, personal and family history of malignancy. Information was also collected regarding the symptoms leading to presentation and the route of referral, subsequently classified into either primary care physician or specialist referral. Data regarding investigations completed prior to and after referral were extracted from imaging reports, referral documents and clinical notes.

A diagnosis of provisional CUP was defined as cancer identified on biopsy without an evident primary after completion of recommended standardised investigations as per international guidelines [8, 21]. A revised diagnosis was recorded if a primary site or tissue of origin was documented to be favoured after discussion in a multi-disciplinary meeting and/or review of clinicopathologic or genomic information. Lymphomas, melanomas, neuroendocrine tumours and sarcomas were excluded. The date of diagnosis was the date of histopathology confirming malignancy. Metastatic sites were classified as solitary if there was an isolated metastasis, otherwise single or multiple organ involvement. Lymph nodes were considered a single organ. Patients with CUP were classified as having either a favourable or unfavourable subtype as outlined in European Society of Medical Oncology guidelines [8, 15]. Renal and lung subtypes were included as favourable subtypes as suggested by recent publications [14].

Data regarding genomic testing were obtained from clinical notes and molecular sequencing reports. Genomic testing included either gene-expression assays for tissue of origin testing, or next-generation DNA sequencing looking for targetable alterations. The impact of genomic testing on patient management was recorded where available, for example identification of a primary tumour, targetable molecular alteration or pathogenic germline variant. Treatment and outcome data for patients with a diagnosis of CUP were recorded where available.

### Statistical analysis

Descriptive statistics (percentages and medians) were used to report patient and disease characteristics, diagnostic work-up and treatments delivered. OS was analysed using the Kaplan–Meier method and defined as months from histological diagnosis of CUP to death, censored at the last visit date. Subgroup differences were compared using the log-rank test, where a *p*-value of <0.05 was considered statistically significant. Statistical analyses were performed using Stata version 12 for Windows, StataCorp. 2011, College Station, TX, USA.

## Results

### Demographics and clinical characteristics

Our cohort consisted of 361 patients. Patient demographics and clinical characteristics are summarised in Table 1. Median age was 62 years, 53% were female, 84% had an ECOG performance status of 0 or 1 and 91% had a Charlson comorbidity score of less than 3. The most common presenting symptoms were abdominal pain or distension, a palpable lymph node or mass, and back pain, accounting for ~50% of cases, collectively. Incidental findings led to referral in 7% of all cases. Metastases to multiple organs were identified in 62% of all patients. A solitary lesion was identified in 36 patients overall (10%), with the location most frequently being bone in 39% (14/36) of cases, followed by a solitary lymph node in 25% (9/36) of cases.

**Table 1:** Characteristics of patients seen in the CUP clinic from 2014–2020.

	All patients (*n* = 361)
Median age (years)	62
Sex	
Female	191 (53%)
Male	170 (47%)
Smoker	
Current	63 (17%)
Ex-smoker	121 (34%)
Never-smoker	151 (42%)
Unknown	26 (7%)
Personal history of cancer	
Yes	103 (29%)
No	258 (71%)
Family history of cancer	
Yes	163 (45%)
No	188 (52%)
Unknown	10 (3%)
Charlson comorbidity index	
0	222 (61%)
1	82 (23%)
2	25 (7%)
3	15 (4%)
4	7 (2%)
≥5	10 (3%)
ECOG performance status at referral	
0	119 (33%)
1	185 (51%)
2	43 (12%)
3	8 (2%)
4	0
Unknown	6 (2%)
Sites of metastases	
Solitary lesion	36 (10%)
Bone only	14 (4%)
Lymph node	9 (2%)
Mass	7 (2%)
Other	6 (2%)
Single organ	88 (24%)
Multiple organs	223 (62%)
Unknown	14 (4%)
Presenting symptoms	
Abdominal pain or distension	65 (18%)
Palpable lymph node or mass	59 (16%)
Back pain	55 (15%)
Systemic symptoms (loss of weight, fatigue, sweats, lethargy)	35 (10%)
Respiratory symptoms	23 (6%)
Chest pain	18 (5%)
Hip/leg pain	16 (4%)
Incidental finding	24 (7%)
Abnormal non-invasive pre-natal blood test	8 (2%)
Other	58 (16%)

*CUP* cancer of unknown primary.

### Referral pattern and diagnostic work-up

Referrals were received equally from primary care physicians and specialists (Table 2). Specialist referrals were received from oncologists in 70% of cases, surgeons in 13%, and other specialists in 17%. The reason for referral from primary care was predominantly for investigation of malignancy of unknown origin (95%), whereas 63% of specialist referrals were for access to genomic testing or clinical trials. The overall rate of completion of full diagnostic work-up as outlined by the European Society for Medical Oncology (ESMO) guidelines [8] was 24% vs 48% for primary care compared to specialist referrals.

**Table 2:** Referral patterns and diagnostic work-up performed.

	Primary care physician (*n* = 182)	Specialist (*n* = 179)
Reason for referral		
Diagnosis (MUO)	173 (95%)	66 (37%)
Sequencing or trial	9 (5%)	113 (63%)
Investigations performed prior to referral		
Baseline bloods	165 (91%)	173 (97%)
CT chest, abdomen, and pelvis	117 (64%)	150 (84%)
Biopsy	69 (38%)	163 (91%)
Mammogram (females only)	30/89 (34%)	38/102 (37%)
PSA (males with bone disease)	20/30 (67%)	17/21 (81%)
Endoscopy	28 (15%)	85 (47%)
PET scan	35 (19%)	116 (65%)
Completed work-up per ESMO clinical practice guidelines [3] prior to referral	43 (24%)	86 (48%)
Investigations performed after referral		
Baseline bloods	102 (56%)	44 (25%)
CT chest, abdomen and pelvis	51 (28%)	15 (8%)
Biopsy (total)	95 (52%)	31 (17%)
Initial biopsy	72 (40%)	10 (5%)
Repeat biopsy	23 (12%)	21 (12%)
PET scan	92 (51%)	47 (26%)

*ESMO* European Society of Medical Oncology, *MUO* malignancy of unknown origin, *PET* positron emission tomography, *PSA* prostate specific antigen.

Following review in the CUP clinic, a full restaging CTCAP was required in 18% of all patients while completion of staging was required in 4%. A PET scan was requested in 39% and MRI scan in 8%. A biopsy was required in 35% of all patients, including 12% of patients who had a biopsy performed prior to referral but required a repeat biopsy to establish a diagnosis or perform genomic testing.

### Diagnosis and CUP classification

**Fig. 1:**
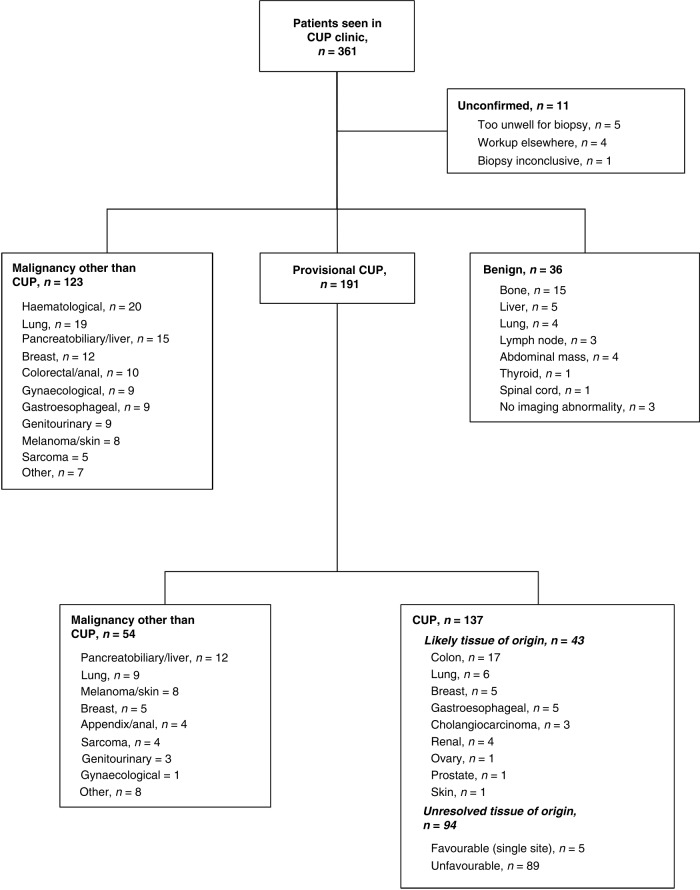
CONSORT diagram of patients seen in the Cancer of Unknown Primary (CUP) clinic. Diagnostic outcomes are described for all patients following completion of standard investigations. For patients with provisional CUP, diagnosis was updated following discussion at a multi-disciplinary meeting and/or review of clinicopathologic or genomic information.

Following completion of standard investigations, 191 patients had a diagnosis of provisional CUP, 123 had a malignancy other than CUP (6 were recurrences of a prior malignancy) and 36 had benign pathologies, of which 9 were biopsy-proven (Fig. 1). Diagnosis was unconfirmed in 11 patients. Of the patients with a provisional CUP diagnosis, 89 (47%) were discussed at a disease-specific multi-disciplinary meeting (MDM) and 118 (62%) had genomic testing performed. Gene-expression tissue of origin testing was completed in 58 patients and comprehensive cancer gene-panel DNA sequencing was completed in 112 patients; 55 patients had both tests successfully completed. Seventy (37%) patients did not undergo genomic testing due to insufficient tissue (*n* = 33), diagnosis resolved based on clinicopathologic features (*n* = 14), clinical deterioration (*n* = 13), testing not available (*n* = 4), testing declined (*n* = 3), loss to follow-up (*n* = 2) and lost sample (*n* = 1). Three further patients had genomic testing requested but no information was found on whether this was successfully completed.

Following MDM discussion and/or second review of histology and genomic results, 28% (54/191) of patients had a documented change of diagnosis to malignancy other than CUP, supported by genomic findings in 21 cases and based on clinicopathologic findings alone in 33 cases. Thirteen cases were determined to be recurrences of prior cancers. A further 43 patients had CUP with a favoured tissue of origin (35 of favourable subtype), supported by genomic findings in 28 cases and based on clinicopathologic findings alone in 15 cases. The remaining 94 patients had CUP with an unresolved tissue of origin, 5 of whom had a favourable subtype due to single-site involvement. The total number of CUP patients with a favourable subtype was 40/137 (29%). Further details on histopathologic subtypes are provided in Fig. 1. Two patients also had incidental pathogenic germline variants identified on genomic testing.

### Treatment and outcomes of patients with CUP

Treatment information was available for 120/137 (88%) patients with CUP. One hundred patients (83%) received systemic therapy: 54 received empirical chemotherapy only and 46 received site-specific therapy (*n* = 20), targeted therapy (*n* = 14) or immunotherapy (*n* = 12) during their treatment course. Patients who received site-specific, targeted therapy or immunotherapy were more likely to have a predicted tissue of origin (67% vs 13%, *p* < 0.0001) or favourable subtype (61% vs 9%, *p* < 0.0001) compared to those who received empirical chemotherapy only (Table 3). Of the patients who received targeted therapy, 9 were treated according to a molecular alteration identified through genomic testing, four received treatment as part of site-specific therapy and one patient initially received pazopanib for a suspected renal primary but subsequently received chemotherapy for a likely gynaecological malignancy based on genomic findings (Table 4). Two patients who received immunotherapy had high TMB or mismatch repair deficiency, respectively.

**Table 3:** Characteristics of CUP patients who received systemic therapy.

	All CUP patients who received systemic therapy (*n* = 100)	Empirical chemotherapy only (*n* = 54)	Site-specific, targeted therapy or immunotherapy (*n* = 46)
Median age (years)	63	63	63
Sex			
Female	54	31 (57%)	23 (50%)
Male	46	23 (43%)	23 (50%
Charlson comorbidity index			
<3	94	50 (93%)	44 (96%)
≥3	6	4 (7%)	2 (4%)
ECOG performance status			
0–1	88	47 (87%)	41 (89%)
≥2	10	6 (11%)	4 (9%)
Unknown	2	1 (2%)	1 (2%)
Likely tissue of origin			
Yes	38	7 (13%)	31 (67%)
No	62	47 (87%)	15 (33%)
Favourable subtype			
Yes	33	5 (9%)	28 (61%)
No	67	49 (91%)	18 (39%)

*CUP* cancer of unknown primary, *ECOG* Eastern Cooperative Oncology Group.

**Table 4:** Demographics, CUP classification and genomic results of 14 patients treated with targeted therapy.

Age	Sex	CUP subtype	CUP subtype detail	Molecular target identified	Molecular target detail	Targeted therapy
60	M	Favourable	Renal-like	No	—	Pazopanib
59	M	Favourable	Lung-like	Yes	*EGFR* exon 18 mutation	Erlotinib
63	M	Favourable	Colon-like	No	—	Regorafenib
56	F	Favourable	Breast-like	Yes	*ERBB2* amplification	Trastuzumab and pertuzumab
73	F	Favourable	Breast-like	Yes	*ERBB2* amplification	Trastuzumab and pertuzumab
60	F	Unfavourable	—	Yes	*MYC* amplification	BET inhibitor
70	M	Unfavourable	—	Yes	*BRCA2* mutation	Talazoparib
64	F	Unfavourable	—	No	—	Pazopanib
69	F	Favourable	Renal-like	No	—	Pazopanib
64	M	Unfavourable	—	Yes	*CDK3* mutation	Ribociclib and trametinib
71	M	Favourable	Renal-like	No	—	Pazopanib
24	F	Unfavourable	—	Yes	*PIK3CA* and *PTEN* alterations	Ipatasertib
48	M	Unfavourable	—	Yes	*BRAF* mutation	Vemurafenib
39	F	Unfavourable	—	Yes	*ERBB2* amplification	Trastuzumab, Ado-trastuzumab emtansine

*BET* Bromodomain and Extra-Terminal motif.

**Fig. 2:**
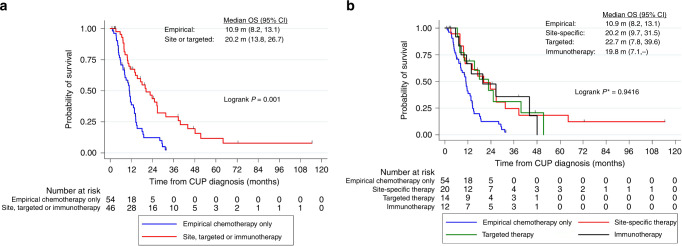
Overall survival of patients with CUP, stratified by type of systemic therapy received. **a** Site-specific, targeted therapy and immunotherapy combined. **b** Site-specific, targeted therapy and immunotherapy presented individually; *p*-value excluding empirical chemotherapy group. OS overall survival.

Overall survival was significantly longer for patients who received site-specific, targeted therapy or immunotherapy (median 20.2 months, 95% CI 13.8–26.7) compared to those who had empirical chemotherapy alone (median 10.9 months, 95% CI 8.2–13.1), with a hazard ratio of 0.31 (95% CI 0.16–0.61, *p*-value = 0.001) after adjustment for age, sex, performance status, Charlson comorbidity score, CUP subtype and if tissue of origin was favoured (Fig. 2a). There was no difference in survival among patients who received site-specific therapy (median 20.2 months, 95% CI 9.7–31.5), targeted therapy (median 22.7 months, 95% CI 7.8–39.6) or immunotherapy (median 19.8 months, 95% CI 7.1–no estimate), log-rank *p*-value = 0.9416 (Fig. 2b). Median OS for patients with favourable and unfavourable subtypes were 23.7 months (95% CI 13.8–31.5) and 10.9 months (95% CI 8.2–13.6), respectively; this includes patients who did not receive systemic therapy.

## Discussion

The CUP clinic at the Peter MacCallum Cancer Centre is the first dedicated CUP clinic in Australia, established to streamline investigations and management of patients with MUO and CUP. Over the course of 6 years, 361 patients were seen in the clinic, with an equal proportion of patients referred from primary care versus other specialists. In patients referred by primary care physicians, a biopsy or completion of staging scans was often all that was required to identify the underlying primary, as most of these patients were referred for management of MUO as opposed to CUP. Adherence to the ESMO guidelines’ diagnostic pathway was higher in patients referred by specialists compared to primary care physicians, consistent with most of these referrals being received from medical oncologists for patients with provisional CUP, for access to genomic testing or trials. The rate of referral for mammograms was low among both groups of referrers (36% of female patients); while this is higher than the 5% rates reported in an older (2006–2011) Australian study [22], it suggests the need for ongoing education about mammography being a recommended part of the work-up for female patients [15]. The low uptake of mammography remains unexplained, but may reflect uncertainty around its diagnostic utility in the setting of advanced imaging modalities such as PET or MRI, as well as conflicting international guidelines, some of which only recommend mammography for clinical presentations that are compatible with breast cancer [9, 10].

Our findings mirror those reported by Stares et al. [23], who described their 10-year experience of the Edinburgh Cancer Centre’s CUP service, with a few notable differences. A substantial proportion of patients (27%) in the Scottish study did not undergo comprehensive investigations for their suspected cancers, whereas a third of patients seen in our clinic had already been diagnosed with provisional CUP after extensive work-up and were referred specifically for genomic testing or clinical trials. This likely reflects the high proportion of inpatient referrals in the Scottish study compared to our study that focused on our outpatient service. Nevertheless, our study demonstrates the dual role of a CUP specialist clinic in facilitating diagnosis and management of patients presenting with MUO and driving research in CUP, both of which are recognised as key priorities in the UK National Institute for Health and Care Excellence guidelines [10]. In particular, we find that second anatomical pathology review, as well as chasing information about prior cancer diagnoses can be very helpful, as 7% of provisional CUP cases in our clinic were subsequently confirmed to be atypical or late recurrences of a prior cancer.

In our study, genomic testing was successfully completed in just over 60% of patients with provisional CUP and impacted management in a substantial proportion of patients by supporting the diagnosis of a primary site or CUP subtype, identifying a molecular alteration for targeted therapy or a germline finding of potential significance. Of note, in half of the cases where a primary site or tissue of origin was favoured, the diagnosis was made based on clinicopathologic criteria alone, highlighting the importance of robust histopathological review and multi-disciplinary input. Nonetheless, in CUP cases that remain unresolved after appropriate immunohistochemical work-up, genomic testing may provide additional diagnostic evidence and sometimes allow a tissue of origin to be determined in cases with atypical immunohistochemistry results for the tumour type [24]. Indeed, the National Comprehensive Cancer Network (NCCN) and ESMO Precision Medicine Working Group recommendations both support the use of genomic testing in patients with a CUP diagnosis [9, 25].

We observed better survival outcomes in our cohort compared to historical reports, particularly in patients with unfavourable CUP where median OS of 10.9 months was almost double the typical 6-month OS in this poor prognosis subgroup [16]. This may reflect improved access to treatments both for anti-cancer therapy and best supportive care; however, selection bias is likely to be a major contributor, as patients referred to our centre for genomic testing or clinical trials are likely to have been fitter or could have already been receiving chemotherapy at the time of referral, while patients who deteriorated rapidly would not have been referred.

Patients who received site-specific therapy, targeted therapy or immunotherapy had longer overall survival compared to those who only received empiric platinum-based chemotherapy. Whilst randomised trial data have not shown a survival advantage for molecularly-directed site-specific therapy over empiric chemotherapy [26, 27], other cohort studies have reported longer overall survival among patients who received molecular-guided therapy [28–30]. The randomised phase II CUPISCO trial (NCT03498521) has recently completed recruitment and will provide further evidence about whether molecularly-directed therapy is superior to platinum-based chemotherapy. However, as diagnostic modalities continue to improve and more treatment options become available for many tumour types, it will become increasingly difficult to conduct prospective randomised CUP trials, as more favourable subtypes are likely to be identified. Challenges in identifying eligible patients for the CUPISCO study through standardised screening have already been highlighted by the study team, with a substantial proportion of patients screen failed as unfavourable CUP could not be confirmed on central review [31].

Our study has several limitations that limit the generalisability of our findings. The patients in our cohort were relatively fit (84% with ECOG performance status 0–1), with the majority of CUP patients receiving systemic therapy. In contrast, rates of systemic therapy use in other CUP cohorts are typically well below 50% [22, 23, 32]. This likely reflects the patient selection for this study through outpatient clinic bookings and the high proportion of patients who were referred specifically for clinical trials or genomic testing. We elected not to use International Classification of Diseases (ICD) codes to identify all CUP patients seen at our institution, as the purpose of this study was to describe the experience of our CUP specialist clinic. Further, a previous audit of ICD-based CUP diagnoses entered in an Australian cancer registry showed that 30% were subsequently reclassified to a known primary site [33], demonstrating the limitations of CUP classifications in cancer registries and hospital records. Finally, the retrospective nature of this study and patient selection means that the true impact of genomic testing on treatment selection and outcomes among the broader CUP patient population is unknown.

In conclusion, this retrospective study assessing the experience of Australia’s first dedicated CUP clinic has shown that a large proportion of patients underwent comprehensive work-up, including genomic testing. Genomic testing impacted the management of approximately one third of patients with initial provisional CUP, which may have contributed to the overall improvement in outcomes when compared to previously reported literature. This highlights the need to offer streamlined management of patients with CUP and lends weight to the benefits derived from genomic testing of CUP patients.

## Data Availability

The dataset used and/or analysed for this study are available from the corresponding author on reasonable request.

## References

[1] Rassy E, Pavlidis N (2020). Progress in refining the clinical management of cancer of unknown primary in the molecular era. Nat Rev Clin Oncol.

[2] Pentheroudakis G, Golfinopoulos V, Pavlidis N (2007). Switching benchmarks in cancer of unknown primary: from autopsy to microarray. Eur J Cancer.

[3] Australian Institute of Health and Welfare. Cancer in Australia 2019. Cancer series no.119. Cat. no. CAN 123. Canberra: AIHW; 2019.

[4] Kolling S, Ventre F, Geuna E, Milan M, Pisacane A, Boccaccio C (2019). “Metastatic Cancer of Unknown Primary” or “Primary Metastatic Cancer”?. Front Oncol.

[5] Abbruzzese JL, Abbruzzese MC, Hess KR, Raber MN, Lenzi R, Frost P (1994). Unknown primary carcinoma: natural history and prognostic factors in 657 consecutive patients. J Clin Oncol.

[6] Urban D, Rao A, Bressel M, Lawrence YR, Mileshkin L (2013). Cancer of unknown primary: a population-based analysis of temporal change and socioeconomic disparities. Br J Cancer.

[7] Samadder NJ, Smith KR, Hanson H, Pimentel R, Wong J, Boucher K (2016). Familial risk in patients with carcinoma of unknown primary. JAMA Oncol.

[8] Fizazi K, Greco FA, Pavlidis N, Daugaard G, Oien K, Pentheroudakis G (2015). Cancers of unknown primary site: ESMO Clinical Practice Guidelines for diagnosis, treatment and follow-up. Ann Oncol..

[9] National Comprehensive Cancer Network. Occult Primary (Version 1.2022). 2019. https://www.nccn.org/professionals/physician_gls/pdf/occult.pdf. 10.6004/jnccn.2014.0093 24994917

[10] National Institute for Health and Care Excellence (NICE). Metastatic malignant disease of unknown primary origin in adults: diagnosis and management. 2010. https://www.nice.org.uk/guidance/cg104. 31846261

[11] Walker MS, Weinstein L, Luo R, Marino I (2018). Pretreatment costs of care and time to initial treatment for patients with cancer of unknown primary. J Comp Eff Res.

[12] Hyphantis T, Papadimitriou I, Petrakis D, Fountzilas G, Repana D, Assimakopoulos K (2013). Psychiatric manifestations, personality traits and health-related quality of life in cancer of unknown primary site. Psychooncology.

[13] Wagland R, Bracher M, Drosdowsky A, Richardson A, Symons J, Mileshkin L (2017). Differences in experiences of care between patients diagnosed with metastatic cancer of known and unknown primaries: mixed-method findings from the 2013 cancer patient experience survey in England. BMJ Open.

[14] Rassy E, Parent P, Lefort F, Boussios S, Baciarello G, Pavlidis N (2020). New rising entities in cancer of unknown primary: Is there a real therapeutic benefit?. Crit Rev Oncol Hematol.

[15] Kramer A, Bochtler T, Pauli C, Baciarello G, Delorme S, Hemminki K (2023). Cancer of unknown primary: ESMO Clinical Practice Guideline for diagnosis, treatment and follow-up. Ann Oncol.

[16] Pavlidis N, Pentheroudakis G (2012). Cancer of unknown primary site. Lancet..

[17] Tothill RW, Li J, Mileshkin L, Doig K, Siganakis T, Cowin P (2013). Massively-parallel sequencing assists the diagnosis and guided treatment of cancers of unknown primary. J Pathol.

[18] Ross JS, Sokol ES, Moch H, Mileshkin L, Baciarello G, Losa F (2021). Comprehensive genomic profiling of carcinoma of unknown primary origin: retrospective molecular classification considering the CUPISCO study design. Oncologist.

[19] Mileshkin LR, Sivakumaran T, Etemadmoghadam D, Tothill R, Fellowes A, Fox SB (2019). Clinical impact of tissue of origin testing and mutation profiling in the Solving Unknown Primary Cancer (SUPER) national prospective study: experience of the first two years. J Clin Oncol.

[20] Charlson ME, Pompei P, Ales KL, MacKenzie CR (1987). A new method of classifying prognostic comorbidity in longitudinal studies: development and validation. J Chronic Dis.

[21] Cancer Council Australia. Optimal care pathway for people with cancer of unknown primary. 2020. https://www.cancer.org.au/assets/pdf/cancer-of-unknown-primary-january-2020.

[22] Tan SYS, O’Neill S, Goldstein D, Ward RL, Daniels B, Vajdic CM (2018). Predictors of care for patients with cancer of unknown primary site in three Australian hospitals. Asia Pac J Clin Oncol.

[23] Stares M, Purshouse K, Knowles G, Haigh R, Irvine J, Gatenby A (2021). Characterisation and outcomes of patients referred to a regional cancer of unknown primary team: a 10-year analysis. Br J Cancer.

[24] Posner A, Prall OW, Sivakumaran T, Etemadamoghadam D, Thio N, Pattison A (2023). A comparison of DNA sequencing and gene expression profiling to assist tissue of origin diagnosis in cancer of unknown primary. J Pathol.

[25] Mosele F, Remon J, Mateo J, Westphalen CB, Barlesi F, Lolkema MP (2020). Recommendations for the use of next-generation sequencing (NGS) for patients with metastatic cancers: a report from the ESMO Precision Medicine Working Group. Ann Oncol.

[26] Hayashi H, Kurata T, Takiguchi Y, Arai M, Takeda K, Akiyoshi K (2019). Randomized phase II trial comparing site-specific treatment based on gene expression profiling with carboplatin and paclitaxel for patients with cancer of unknown primary site. J Clin Oncol.

[27] Fizazi K, Maillard A, Penel N, Baciarello G, Allouache D, Daugaard G (2019). A phase III trial of empiric chemotherapy with cisplatin and gemcitabine or systemic treatment tailored by molecular gene expression analysis in patients with carcinomas of an unknown primary (CUP) site (GEFCAPI 04). Ann Oncol.

[28] Hainsworth JD, Rubin MS, Spigel DR, Boccia RV, Raby S, Quinn R (2013). Molecular gene expression profiling to predict the tissue of origin and direct site-specific therapy in patients with carcinoma of unknown primary site: a prospective trial of the Sarah Cannon research institute. J Clin Oncol.

[29] Moran S, Martinez-Cardus A, Sayols S, Musulen E, Balana C, Estival-Gonzalez A (2016). Epigenetic profiling to classify cancer of unknown primary: a multicentre, retrospective analysis. Lancet Oncol.

[30] Fusco MJ, Knepper TC, Balliu J, Del Cueto A, Laborde JM, Hooda SM (2022). Evaluation of targeted next-generation sequencing for the management of patients diagnosed with a cancer of unknown primary. Oncologist.

[31] Pauli C, Bochtler T, Mileshkin L, Baciarello G, Losa F, Ross JS (2021). A Challenging Task: Identifying Patients with Cancer of Unknown Primary (CUP) According to ESMO Guidelines: The CUPISCO Trial Experience. Oncologist.

[32] Mileshkin L, Bochtler T, Gatta G, Kurzrock R, Beringer A, Muller-Ohldach M (2022). Cancer-of-Unknown-Primary-Origin: A SEER-Medicare Study of Patterns of Care and Outcomes among Elderly Patients in Clinical Practice. Cancers (Basel).

[33] Vajdic CM, Er CC, Schaffer A, Dobbins T, Wyld L, Meagher NS (2014). An audit of cancer of unknown primary notifications: a cautionary tale for population health research using cancer registry data. Cancer Epidemiol.

